# Pyogenic Liver Abscess as a Cause of Sepsis: A Case Report

**DOI:** 10.7759/cureus.81733

**Published:** 2025-04-04

**Authors:** Ricardo Morales-Garcia, Victor M Ayuso-Diaz, Iván Sanchez, Nayeli X Rico-Sanchez, Karen V Patiño-Amaro, Angelica Moreno-Enriquez

**Affiliations:** 1 Medicine, Universidad Nacional Autónoma de México, Mexico City, MEX; 2 Research and Education Division, Medical Care and Research, Yucatán, MEX; 3 Surgery, Elvia Carrillo Puerto Regional Hospital, Yucatan, MEX; 4 Genomic-Metabolic Unit, Marista University of Merida, Yucatan, MEX; 5 Medicine, Universidad Justo Sierra, Mexico City, MEX; 6 Genomic-Metabolic Unit, Marista University of Merida, Yucatán, MEX

**Keywords:** fever of unknown origin, percutaneous drainage, procalcitonin, pyogenic liver abscess, septic evolution, septic shock

## Abstract

Pyogenic liver abscess (PLA) is a potentially life-threatening condition that can progress to sepsis if not treated promptly. The condition often presents with non-specific symptoms such as persistent fever and abdominal pain, which can delay diagnosis. Computed tomography plays a crucial role in identifying hepatic abscesses and guiding timely intervention. We report the case of a 54-year-old man with a one-week history of high fever and epigastric pain. Imaging confirmed the presence of a liver abscess, and due to haemodynamic instability, surgical drainage was performed, yielding purulent material in which *Streptococcus anginosus* was isolated. Subsequent drainage 72 hours later resulted in the complete resolution of the abscess and the successful completion of antibiotic therapy.

## Introduction

Pyogenic liver abscesses (PLAs) are collections of purulent material within the liver parenchyma resulting from the invasion of microorganisms into a previously sterile site. Common pathogens include *Escherichia coli*, *Klebsiella pneumoniae*, *Streptococcus* spp., and *Enterococcus* spp. [1,2]. Although rare, liver abscesses are clinically important because they can rapidly progress to sepsis if not diagnosed and treated promptly [3,4]. In certain clinical scenarios, patients presenting with fever of unknown origin (FUO) may ultimately be diagnosed with a PLA, making it a rare but important consideration in the differential diagnosis of FUO.

The incidence of PLA varies geographically, ranging from 2.3 to 17.59 cases per 100,000 population per year [1,2,3]. Risk factors include intravenous drug use, immunosuppression, diabetes mellitus, use of proton pump inhibitors, bacteraemia of extrahepatic origin, cirrhosis, and a history of organ transplantation or splenectomy [3,5,6].

Early clinical manifestations of liver abscess typically include fever, right upper quadrant or epigastric pain, anorexia, nausea, and vomiting [2,7,8]. The diagnostic workup usually includes a combination of laboratory investigations and imaging studies. Ultrasound and contrast-enhanced computed tomography (CT) scans are critical in detecting purulent collections and differentiating them from other hepatic pathologies [5,9,10]. Prompt treatment with broad-spectrum antibiotics combined with appropriate drainage - whether percutaneous or surgical - is essential to prevent complications such as septic shock and multi-organ failure [4,11,12].

By acknowledging that a subset of patients with FUO may have an underlying liver infection, clinicians are reminded of the importance of maintaining a broad differential diagnosis. This approach is critical to the initiation of timely and targeted therapeutic interventions, ultimately improving patient outcomes.

## Case presentation

A 54-year-old man, with a history of systemic arterial hypertension treated with amlodipine 5 mg/valsartan 160 mg daily, presented with a seven-day history of intermittent, unquantified fever and nocturnal diaphoresis. Initially, he self-medicated with paracetamol and over-the-counter antihistamines, believing that his symptoms were due to an upper respiratory tract infection. However, due to persistent fever and the onset of non-radiating, burning epigastric pain accompanied by diaphoresis, he sought care at the emergency department.

On presentation, vital signs revealed hypotension (89/68 mmHg), tachycardia (123 bpm), tachypnoea (22 breaths per minute), fever (38.7°C), and reduced oxygen saturation (86% on room air). The patient was alert and oriented, with preserved neurological function but evident dehydration. The cardiopulmonary examination was unremarkable. Abdominal palpation elicited moderate to deep epigastric tenderness without signs of peritoneal irritation. The extremities were free of oedema, but capillary refill was delayed to four seconds, indicating hypoperfusion.

Initial laboratory tests demonstrated leukocytosis with neutrophilia, mild transaminitis, elevated bilirubin levels, hyponatraemia, and a markedly raised procalcitonin level, all suggesting an underlying systemic infection (Table 1).

**Table 1:** Evolution of laboratory parameters

Parameter	On admission	Post-treatment	Reference range
White blood cells (WBC)	17.0 × 10⁹/L	7.8 × 10⁹/L	4.0-11.0 × 10⁹/L
Neutrophils	13.7 × 10⁹/L	5.9 × 10⁹/L	1.5-8.0 × 10⁹/L
Total bilirubin	1.5 mg/dL	0.7 mg/dL	0.3-1.2 mg/dL
Aspartate transaminase (AST)	66 U/L	32 U/L	10-40 U/L
Alanine transaminase (ALT)	84 U/L	41 U/L	7-56 U/L
Procalcitonin	2.5 ng/mL	<0.1 ng/mL	<0.5 ng/mL

**Figure 1:**
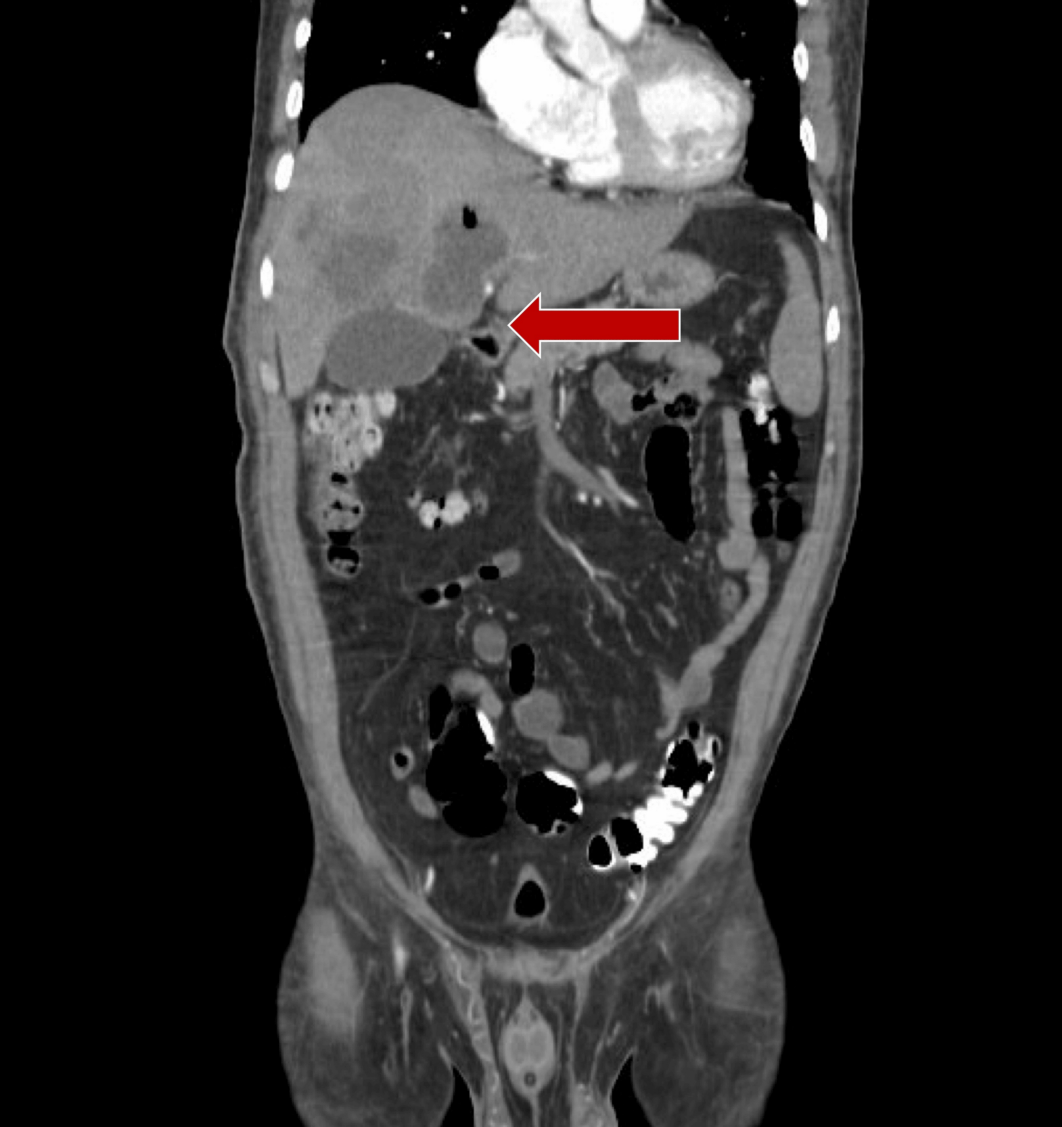
Abdominal CT scan Red arrow: liver lesion with irregular contours and heterogeneous parenchyma, characterized by hypodense areas (16 HU) interspersed with areas of extremely low density (-832 HU), internally divided by septa and accompanied by perilesional edema. The specimen measured approximately 112 × 109 × 104 mm.

The mild elevation of bilirubin and hepatic enzymes was attributed to systemic inflammatory response syndrome (SIRS) and hepatic congestion due to sepsis rather than direct hepatocellular injury. Initial management included proton pump inhibitors and antispasmodics, and the patient was admitted for a diagnostic protocol targeting febrile illness of possible infectious origin. A contrast-enhanced abdominal CT scan identified a heterogeneous hepatic lesion with characteristics suggestive of an infectious process, localized to segments IVa, IVb, VI, and VII, with surrounding edema (Figure 1).

**Figure 2:**
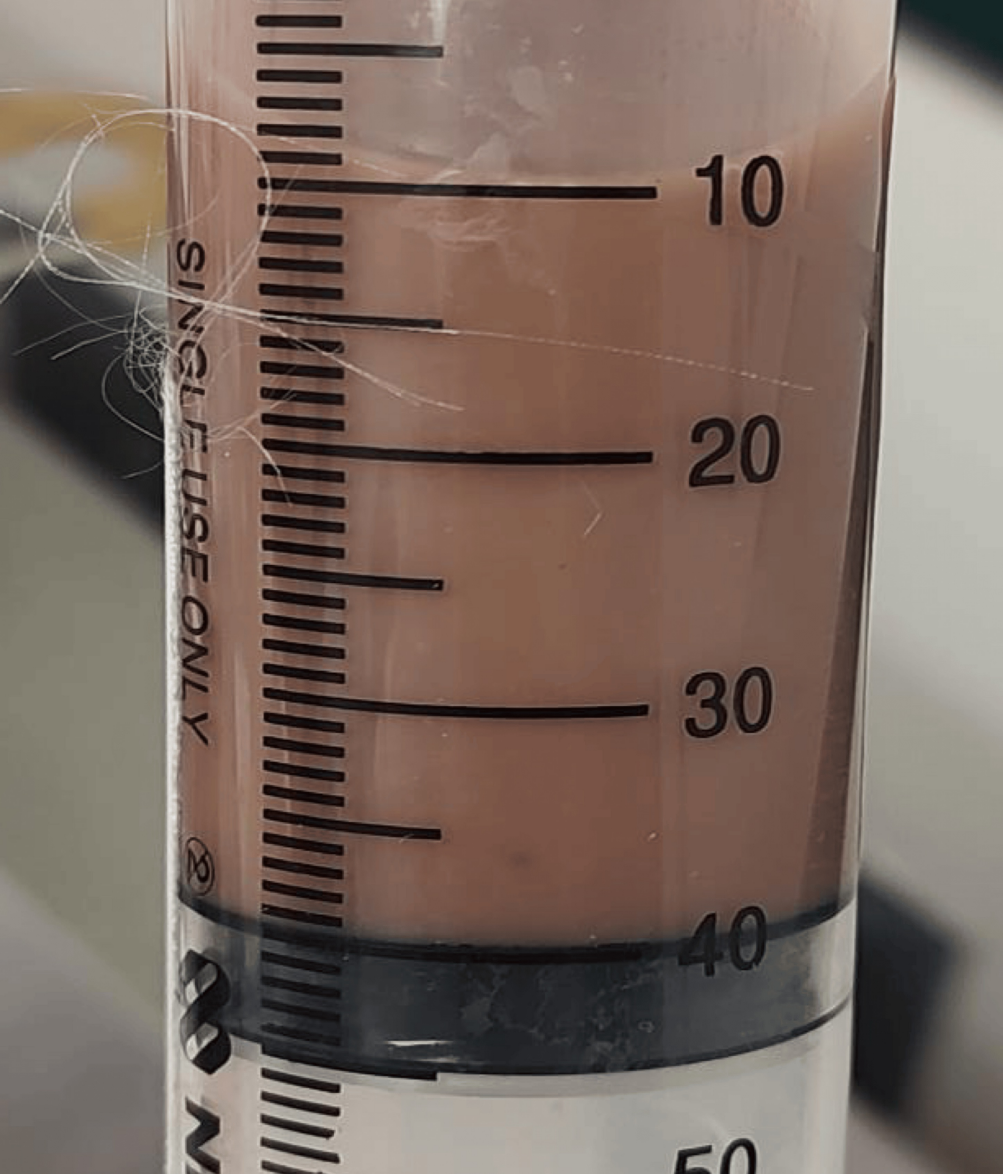
Drainage of liver abscess in segments V and VI 96 ml of purulent material with a characteristic "anchovy broth" appearance.

Empirical antibiotic therapy was initiated with metronidazole 750 mg IV every eight hours and ceftriaxone 1 g IV every 12 hours. However, the patient experienced sudden haemodynamic collapse with a mean arterial pressure of 40 mmHg, tachycardia (120 bpm), and a fever of 39.2°C. A central venous catheter was inserted for vasoactive support, and the general surgery team was consulted. During surgery, purulent material suggestive of amoebic aetiology was evacuated (Figure 2), and a sample was sent for microbiological analysis.

**Figure 3:**
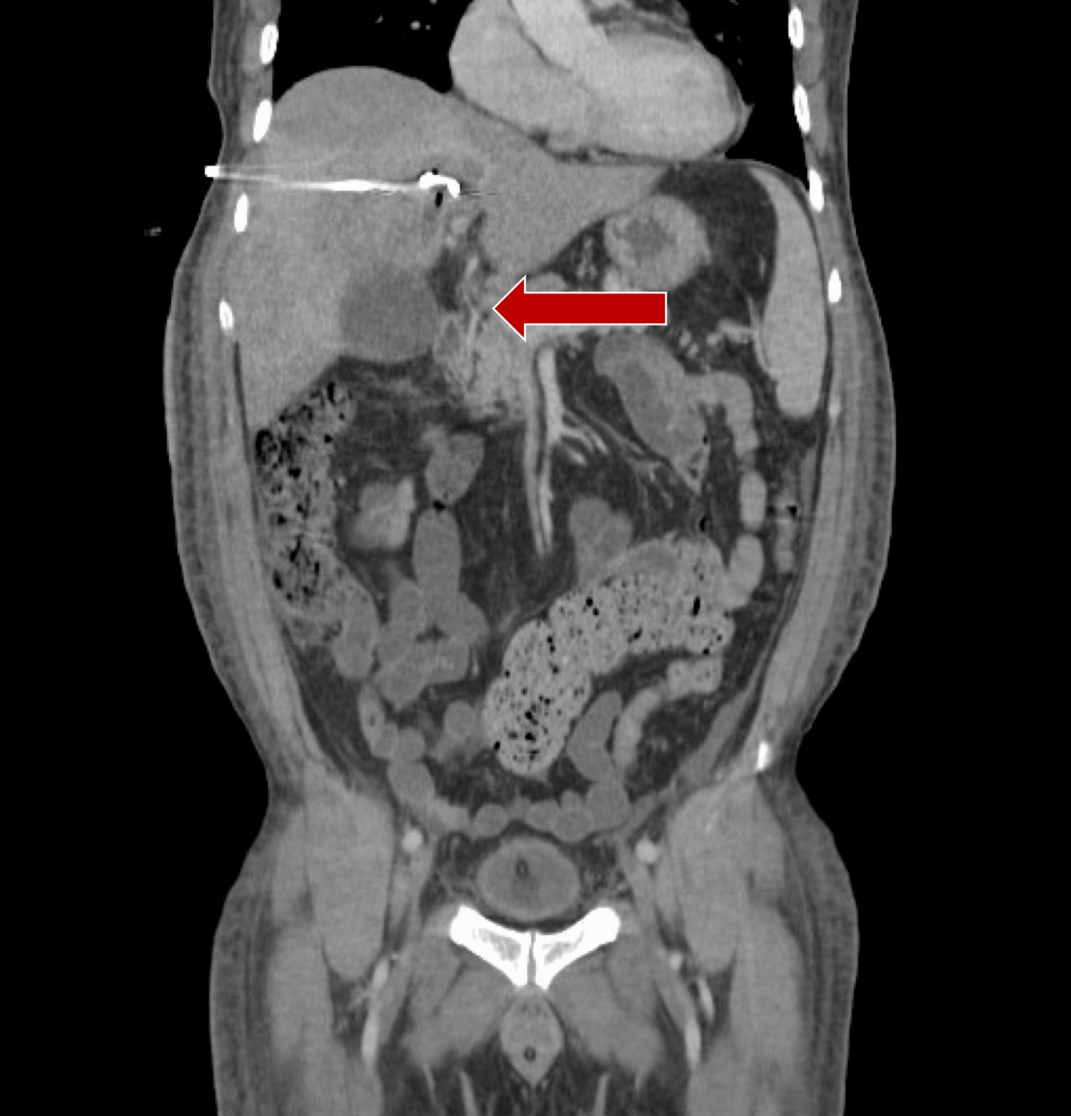
Control CT scan after the second percutaneous drainage Reduction of the liver mass to 31 × 29 × 37 mm, located in segments IVa, IVb, VI and VII, and with drainage in correct position.

Despite the suggestive macroscopic appearance, the microbiological culture of the abscess fluid confirmed the presence of Streptococcus anginosus, a facultative anaerobe commonly associated with pyogenic abscess formation. Given the identification of* Streptococcus anginosus*, and considering its known antibiotic susceptibility profile, the antimicrobial regimen was escalated to piperacillin-tazobactam 4.5 g IV every six hours. A thorough investigation into the possible portal of entry, including dental evaluation and gastrointestinal imaging, was performed but failed to reveal a definitive source. Despite initial drainage, residual collection was evident on follow-up imaging, prompting a second percutaneous drainage, which resulted in the aspiration of additional purulent material and a marked reduction in abscess size (Figure 3).

Reduction of the hepatic abscess to 31 × 29 × 37 mm in segments IVa, IVb, VI, and VII, with drain in optimal position and signs of resolution. The clinical course was favorable. Inflammatory markers progressively normalised (as shown in Table 1), and imaging confirmed complete resolution of the hepatic enhancement. After seven days of targeted antibiotic therapy and sustained clinical improvement, the drainage catheter was safely removed. The patient was discharged in stable condition with scheduled outpatient follow-up. No evidence of recurrence was noted on subsequent imaging.

## Discussion

PLA is a rare but potentially serious condition that can be associated with serious complications and high mortality if not diagnosed and treated in time. Its incidence has shown an increase in recent decades, especially in populations with a high prevalence of metabolic and hepatobiliary diseases, which has been attributed to epidemiological factors and improvements in diagnostic techniques and the identification of emerging aetiological agents [1,2].

This case highlights the importance of considering PLA in the differential diagnosis of patients with prolonged fever and abdominal pain, even in those without obvious risk factors. Although classically associated with biliary disease, diverticulitis, or intra-abdominal sepsis, in some cases, the portal of entry may not be obvious, complicating diagnosis and delaying treatment, thereby increasing the risk of complications [3,4]. Microbiologically, *S. anginosus *is a rare pathogen in liver abscesses, although it is recognized as a microorganism with a marked capacity for tissue invasion and deep abscess formation. Its involvement in monomicrobial and polymicrobial infections has been documented, usually in combination with anaerobes and gram-negative bacilli [5,6]. Its identification in this case reinforces the importance of obtaining adequate cultures and using antimicrobial susceptibility testing to optimise treatment.

The diagnosis of PLA remains a clinical challenge due to its non-specific presentation. Contrast-enhanced computed tomography is considered the gold standard, with a sensitivity approaching 100% in the detection of hepatic collections, surpassing the utility of ultrasonography, which may have limitations in small or deep abscesses [7,8]. In the case described, the imaging findings were essential to guide the therapeutic approach and assess the response to treatment. The treatment of this pathology requires a multidisciplinary approach combining antibiotic therapy and drainage of the collection. It is recommended to start a broad-spectrum empirical therapy covering gram-negative and anaerobic bacilli, adjusting the schedule according to the microbiological findings [9]. In this patient, ceftriaxone and metronidazole were initially started, which is considered an appropriate regimen in most cases; however, following isolation of *S. anginosus*, it was decided to escalate to piperacillin-tazobactam, following the recommendations in the literature for severe infections with this pathogen [10].

Percutaneous drainage is the preferred method in most cases, although surgical intervention may be required in large, septated abscesses or patients with haemodynamic instability [11,12]. In this case, surgical drainage was performed first, followed by percutaneous drainage, reflecting the complexity of the liver collection and the need for a sequential approach to optimize the resolution of the infectious process. From an evolutionary perspective, the therapeutic response was evident with clinical improvement, normalization of inflammatory reactants, and resolution of the liver collection on control imaging studies. These results confirm the efficacy of the therapeutic approach used and emphasise the importance of clinical and radiological monitoring in therapeutic decision-making [13].

## Conclusions

This case highlights the importance of considering PLA in patients presenting with acute febrile illness and abdominal pain, even in the absence of classic risk factors such as immunosuppression or hepatobiliary disease. Although fever was the initial symptom, the clinical picture evolved into sepsis secondary to liver abscess, requiring prompt recognition and escalation of care. Early diagnosis by imaging and microbiology is essential to identify the causative organism and guide effective treatment. Timely sepsis management - including early fluid resuscitation, hemodynamic support, and source control through antibiotic therapy and percutaneous or surgical drainage - is critical to reduce morbidity and mortality.

In this case, *S. anginosus *was isolated, confirming its role as a relevant pathogen in hepatic abscesses and the importance of including it in the differential diagnosis. Furthermore, the patient's favourable outcome illustrates the value of a structured, multidisciplinary approach. Close follow-up is essential to ensure complete resolution and to prevent recurrence. Overall, this case highlights the need for a high index of suspicion and rapid diagnostic and therapeutic intervention in atypical presentations of intra-abdominal sepsis.
